# Addressing tuberculosis in differentiated care provision for people living with HIV

**DOI:** 10.2471/BLT.16.187021

**Published:** 2017-01-01

**Authors:** Ishani Pathmanathan, Eric Pevzner, Joseph Cavanaugh, Lisa Nelson

**Affiliations:** a Division of Global HIV and TB, Centers for Disease Control and Prevention, 1600 Clifton Road, Atlanta, Mailbox E-04, GA, 30329, United States of America (USA).; b Office of the Global AIDS Coordinator, Washington, USA.

Despite advances in prevention, diagnosis and treatment of tuberculosis and human immunodeficiency virus (HIV), tuberculosis remains the leading cause of death and illness among people living with HIV. In 2015, an estimated 1.2 million of the people who developed tuberculosis disease worldwide were HIV positive, and tuberculosis was the direct cause of at least one third of HIV-related deaths.^1^ The 2015 “Treat All” strategy requires that everyone with HIV is offered antiretroviral therapy (ART) as soon as they are diagnosed. By treating HIV infections earlier, this strategy should mitigate the HIV-associated tuberculosis epidemic, but it alone is not sufficient to eliminate preventable tuberculosis suffering and deaths among people living with HIV.^2^ The 2016 World Health Organization (WHO) guidelines recommend differentiated HIV service delivery, which is intended to facilitate the “Treat All” strategy by tailoring services to the differing needs of individuals.^3^ As HIV programmes adopt these WHO guidelines, tuberculosis also needs to be addressed.^3^

Compared to the general population, people living with HIV have a significantly higher risk of tuberculosis even if they are stable on treatment and have high CD4+ T-lymphocyte counts.^4^ Therefore, WHO recommends that all people living with HIV are screened for tuberculosis symptoms (cough of any duration, weight loss, fever or night sweats) at every patient encounter.^3^ One of the implications of differentiated care is that the intervals between clinic visits and/or antiretroviral pick-ups from pharmacies may be extended to three or six months for people who are stable on ART. However, routine tuberculosis screening is still needed regularly, followed by appropriate evaluation, accurate diagnosis and treatment for either tuberculosis disease or latent infection.^3^ Patients should also be able to receive tuberculosis preventive therapy at the same time that they pick-up their antiretroviral medications.^5^^–^^7^

As part of differentiated care, community health workers, ART clubs and other models of community service delivery are increasingly being used by HIV-treatment programmes. In these models, participants could be trained to screen for symptoms of tuberculosis and other opportunistic infections, refer people for further evaluation and dispense tuberculosis preventive therapy. This approach has been piloted in several sub-Saharan African settings.^8^^–^^12^

In addition, improving participants’ understanding of tuberculosis will help them to recognize symptoms in themselves and family members and advocate for their own care. Active tuberculosis case-finding among household members and close contacts of tuberculosis patients is critical to finding an estimated 4.3 million people with undiagnosed tuberculosis.^1^ More collaboration with national tuberculosis programmes is needed to support or expand community- or household-level tuberculosis case-finding activities, such as sputum collection and transport, directly observed therapy, monitoring for adverse events, tracing of people not completing treatment, as well as nutritional support, and health education.^8^^–^^12^ Collaboration with civil society organizations will also be needed to understand – and adapt service delivery to – local context and needs.

Differentiated care is also expected to make more facility-based resources available to care for patients with advanced and/or unstable disease. For these patients, in addition to the early antiretroviral treatment they should already be receiving, tuberculosis prevention, targeted case-finding and diagnosis are urgently needed. In addition to symptom-screening and rapid diagnostic tests for HIV-associated tuberculosis, supplemental urine lipoarabinomannan testing can be used to diagnose disseminated tuberculosis in the sickest people living with HIV, who are at greatest risk of death.^3^ As stable patients are managed more in communities, and facilities increasingly become service delivery points for patients with advanced disease, strong infection prevention and control measures are required to prevent iatrogenic transmission of tuberculosis.

Integrating tuberculosis and HIV activities will allow more people to benefit from the lifesaving potential of the “Treat All” strategy. Differentiated care-delivery systems that account for both tuberculosis and HIV could improve access to services, use existing human resources and supply chains more efficiently and strengthen monitoring and evaluation efforts for both diseases.
